# Protocol for a systematic review and meta-analysis of data from preclinical studies employing forced swimming test: an update

**DOI:** 10.1136/bmjos-2017-000043

**Published:** 2019-05-31

**Authors:** A B Ramos-Hryb, Z Bahor, S McCann, E Sena, M R MacLeod, C Lino de Oliveira

**Affiliations:** 1 Physiological Sciences Deptartment, Biological Sciences Center, Federal University of Santa Catarina, Florianópolis, Brazil; 2 Centre for Clinical Brain Sciences, University of Edinburgh, Edinburgh, UK

## Abstract

**Objective:**

Forced swimming test (FST) in rodents is a widely used behavioural test for screening antidepressants in preclinical research. Translational value of preclinical studies may be improved by appraisal of the quality of experimental design and risk of biases, which remains to be addressed for FST. The present protocol of a systematic review with meta-analysis aims to investigate the quality of preclinical studies employing FST to identify risks of bias in future publications. In addition, this protocol will help to determine the effect sizes (ES) for primary and secondary outcomes according to several aspects of the FST study design.

**Search strategy, Screening annotation, Data management:**

Publications reporting studies testing different classes of antidepressants in FST will be collected from Medline, SCOPUS and Web of Science databases. A broad list of inclusion criteria will be applied excluding those studies whereby FST is used as a stressor or studies reporting data from co-treatments. For assessing the quality of the included publications, the quality checklist adapted by Collaborative Approach to Meta-Analysis and Review of Animal Data from Experimental Studies will be used. If the meta-analysis seems feasible, the ES and the 95% CI will be analysed. The heterogeneity between studies will be assessed by using the χ^2^statistic with n−1 degrees of freedom. Subgroup meta-analysis (meta-regression, and if necessary, stratified regression) will be performed when possible according to characteristics of study design and study quality to assess their impact on efficacy of the treatments. In addition, funnel plotting, Egger regression, and ‘trim and fill’ will be used to assess the risk of publication bias. Results of this protocol will help to create rational methodological guidelines for application of FST in rodents and improve the quality and translational value of preclinical research on antidepressant discovery.

**Reporting:**

A preliminary version of the present protocol has been preregistered with Systematic Review Facility (http://syrf.org.uk/). A preprint version of the current protocol has been registered with Open Science Framework (https://osf.io/9kxm4/). Results will be communicated in scientific meetings and peer-reviewed journals. We plan to conduct an anonymous and online survey within the scientific community to ask researchers about their perception of risk of bias and their experience with the publication of negative results.

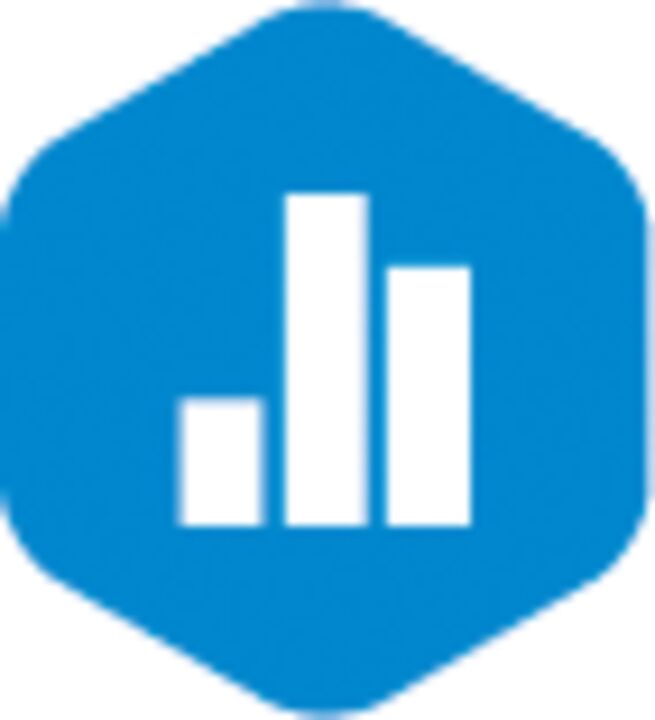

**Strengths and limitations of this study**

- This protocol for systematic review will collect, with broad inclusion criteria, preclinical studies employing forced swimming test (FST).
- The present protocol has been preregistered with Open Science Framework.
- A preliminary version of the present protocol has been preregistered with Systematic Review Facility (Collaborative Approach to Meta-Analysis and Review of Animal Data from Experimental Studies).
- Results obtained with this systematic review and meta-analysis may help to create specific and rational methodological guidelines for application of FST in rodents.
- High levels of heterogeneity between studies may limit the external validity of our results.
- The summary effect size may be overestimated by publication bias.

## Introduction

Major depression disorder (MDD) in humans is characterised by depressed mood and behavioural inhibition and often comes with social avoidance, generalised anxiety, eating disorders, sleeping and problems to cope with stress.^1^ Despite the difficulty in finding suitable models to mimic subjective, behavioural and neurobiological aspects of MDD, there are several animal models predictive of MDD treatment.^2 3^ Most of these animal models are based on behavioural responses of an animal to inescapable stress, providing a framework for several laboratory tests.^4–7^ Usually, inescapable stress induces behavioural inhibition (or immobility) that can be counteracted by antidepressant treatment.^4–7^ Therefore, behavioural tests in animals are employed as screening steps during the preclinical phase of antidepressant drug discovery. Forced swimming test (FST)^4^ in rats and mice is used in preclinical trials of antidepressants. FST is easy to run, inexpensive, sensitive and relatively selective to known antidepressants (for review see Cryan *et al* 2). One criticism that may apply to FST is the abundance of ‘positive results'^8^ that contrasts with the failure of antidepressant treatments in some clinical trials or therapeutics.^9–11^ There is an estimation that up to 50% of patients are resistant to the treatment with the antidepressants currently available.^9 10^ Many different reasons may account for the contrasting findings between preclinical and clinical data^11^ including individual variability, poor quality of the studies as well as publication bias. Publication bias in a preclinical field may inflate the estimated effect size (ES)^12 13^ leading to inflated expectations of efficacy in clinical trials, which may explain partially the perceived contrast between fields.^11^ Therefore, the aim of the present study is to evaluate the quality of published literature applying FST to detect effects of the treatment with antidepressants and the risk of bias in this research field.

Initially, a pilot study was performed to create a database and to standardise the methods for a systematic review.^14 15^ This pilot study started with a review in Medline and Embase retrieving more than 7000 publications by using expressions commonly found in the literature such as ‘forced swimming test’ OR ‘forced swim test’ OR ‘Porsolt test' OR ‘fst’. The combination of these with medical subject heading (MeSH) terms related to ‘rodents'^16^ and ‘antidepressants'^17^ retrieved the publications more relevant for the present study. For screening purposes, a database containing bibliographical information from retrieved publications was built. We applied inclusion and exclusion criteria in the screening steps. Forty references, randomly selected from the database, generated 20 references to the pilot study, that is, one reference in every two fitted the inclusion criteria. From the selected literature, parameters were taken to estimate: (1) Quality. (2) ES. (3) Heterogeneity. (4) Publication bias. Most of the studies included in the pilot study were published from 2007 onwards. The quality score scale, adapted from the Collaborative Approach to Meta-Analysis and Review of Animal Data from Experimental Studies (CAMARADES),^18 19^ revealed that 12 studies scored above the median score (mean=09, maximum=14, minimum=04); none of them scored the maximum (18) or the minimum (0) values of the scale. Interestingly, all studies reported ‘species or strain in the title, abstract or full text’ and none reported ‘sample size calculation’ or ‘concealment of treatment allocation’ indicating that year of publication may not influence the quality of the studies. The median scores for studies in rats and mice were equal to the overall median score. Most of the experiments were performed in male animals. There were 34 different experiments: 15 using tricyclic antidepressants, 16 using selective serotonin reuptake inhibitors and 3 using selective norepinephrine reuptake  inhibitors.

In the 34 experiments, there were 96 comparisons between experimental (439–470 animals) and control groups (276–287). In most of the mentioned experiments, a control group was compared with several different experimental groups generating significant and non-significant results in a single experiment. The incidences of significant results for primary and secondary outcomes within the experiments were 88.2% and 84.6%, respectively. Interestingly, the experiments also showing non-significant results for primary outcomes were only 29.4% whereas most of the experiments (92.3%) also reported non-significant results for secondary outcomes. The high number of significant results as compared with the negative ones was also found in another study.^8^ In summary, these preliminary analyses indicated that quality scores will be independent of the publication date, the experimental species as well as the type of antidepressant tested. The differences in sex, strains and ages may be a source of heterogeneity. In addition, it is expected that ‘random allocation to a treatment’, ‘concealment of treatment allocation’ and ‘sample size calculation’ will be neglected in this field of research. Moreover, these interim data suggest the existence of publication bias. Considering the sample used in the pilot study as the representative sample, the screening process may generate enough data (50% of publications in the database will fit inclusion criteria, ie, 2200 publications) for reliable estimation of the quality of the studies, ES, heterogeneity and publication bias in the field.

A preliminary version of this protocol was deposited in the Systematic Review Facility in February 2016.^14 15^ The current version of the protocol was updated based on procedures available in CAMARADES following instructions by de Vries *et al*.^20^ A preprint version of the current text was preregistered with Open Science Framework (osf.io/9kxm4). Meta-analysis will be performed after publication of the protocol in a journal with a peer-review system. The links containing preliminary versions of the protocol will be updated to acknowledge the existence of the final peer-reviewed version.

### Systematic review questions

- What is the quality level of these studies?
- Is there any relationship between quality scores and ES of outcomes reported in preclinical studies employing FST?
- Is there an influence of the study design or the ES in primary or secondary outcomes of these preclinical studies?
- Is there any risk of bias in preclinical studies employing FST for antidepressant research?

## Methods and analysis

### Systematic review in specialised literature

This protocol was formulated using the Systematic Review Center for Laboratory animal Experimentation format.^20^ The search strategy is based on previously reported protocols^17^ and consists of an update from our previous protocol registered in the CAMARADES platform.^14 15^ Medline, SCOPUS and Web of Science will be the databases selected. The search in Medline will be performed using the Pubmed platform (advanced search in http://www.ncbi.nlm.nih.gov/pubmed). The search in SCOPUS and Web of Science will be conducted accessing the ‘Periodicos Coordenação de Aperfeiçoamento de Pessoal de Nível Superior (CAPES)’ platform (advanced search in http://www-periodicos-capes-gov-br.ez46.periodicos.capes.gov.br/) at the Federal University of Santa Catarina. The selection of keywords was based on the different denominations of FST found in the literature (see tables 1–3). We decided to include in the review only the data from studies in rats and mice that are the most common laboratory species submitted to FST using the MeSH terms by Hooijmans *et al*.^16^ The list of antidepressants included in the research was by McCann *et al*.^17^ The relevant period of publication started from 1977 when the first paper was published^4^ to December 2017.

**Table 1:** Keywords for search in the Medline database

‘rodentia’(MeSH Terms) OR mice(Tiab) OR mus(Tiab) OR mouse(Tiab) OR murine(Tiab) OR woodmouse(tiab) OR rats(Tiab) OR rat(Tiab) OR murinae(Tiab) OR muridae(Tiab) OR cottonrat(tiab) OR cottonrats(tiab) OR rodentia(Tiab) OR rodent(Tiab) OR rodents(Tiab)	#1
(((ssri) OR (ssris) OR (selective serotonin reuptake inhibitor) OR (selective serotonin reuptake inhibitors) OR (selective serotonin re-uptake inhibitor) OR (selective serotonin re-uptake inhibitors) OR (selective serotonin-reuptake inhibitors) OR (selective serotonin-reuptake inhibitor) OR (fluoxetine) OR (citalopram) OR (escitalopram) OR (fluvoxamine) OR (paroxetine) OR (sertraline) OR (dapoxetine) OR (snri) OR (ssris) OR (serotonin and norepinephrine reuptake inhibitors) OR (serotonin and norepinephrine reuptake inhibitor) OR (serotonin and norepinephrine re-uptake inhibitors) OR (serotonin and norepinephrine re-uptake inhibitor) OR (serotonin-noradrenaline reuptake inhibitors) OR (serotonin and norepinephrine reuptake inhibitors) OR (serotonin and norepinephrine reuptake inhibitor) OR (serotonin and norepinephrine re-uptake inhibitors) OR (serotonin and norepinephrine re-uptake inhibitor) OR (serotonin-norepinephrine reuptake inhibitors) OR (duloxetine) OR (levomilnacipran) OR (sibutramine) OR (bicifadine) OR (venlafaxine) OR (desvenlafaxine) OR (milnacipran) OR (tramadol) OR (TCA) OR (tcas) OR (tricyclic antidepressant) OR (tricyclic antidepressants) OR (tricyclic anti-depressant) OR (tricyclic anti-depressants) OR (amitriptyline) OR (butriptyline) OR (clomipramine) OR (desipramine) OR (dosulepin) OR (doxepin) OR (imipramine) OR (iprindole) OR (lofepramine) OR (melitracene) OR (nortriptyline) OR (opipramol) OR (protriptyline) OR (trimipramine) OR (sari) OR (saris) OR (serotonin antagonist and reuptake inhibitor) OR (serotonin antagonist and reuptake inhibitors) OR (serotonin antagonist and re-uptake inhibitor) OR (serotonin antagonist and reuptake inhibitors) OR (etoperidone) OR (lorpiprazole) OR (mepiprazole) OR (lubazodone) OR (nefazodone) OR (trazodone) OR (NRI) OR (nris) OR (norepinephrine reuptake inhibitor) OR (norepinephrine reuptake inhibitors) OR (norepinephrine re-uptake inhibitor) OR (norepinephrine re-uptake inhibitors) OR (norepinephrine reuptake inhibitor) OR (norepinephrine reuptake inhibitors) OR (norepinephrine re-uptake inhibitor) OR (norepinephrine re-uptake inhibitors) OR (atomoxetine) OR (reboxetine) OR (viloxazine) OR (ndri) OR (ndri) OR (norepinephrine dopamine reuptake inhibitor) OR (norepinephrine dopamine reuptake inhibitors) OR (norepinephrine dopamine reuptake inhibitor) OR (norepinephrine dopamine reuptake inhibitors) OR (norepinephrine-dopamine reuptake inhibitor) OR (norepinephrine-dopamine reuptake inhibitors) OR (norepinephrine dopamine reuptake inhibitor) OR (norepinephrine-dopamine reuptake inhibitors) OR (norepinephrine and dopamine reuptake inhibitor) OR (norepinephrine and dopamine reuptake inhibitors) OR (norepinephrine and dopamine re-uptake inhibitor) OR (norepinephrine and dopamine re-uptake inhibitors) OR (norepinephrine dopamine reuptake inhibitor) OR (norepinephrine dopamine reuptake inhibitors) OR (norepinephrine dopamine re-uptake inhibitor) OR (norepinephrine dopamine re-uptake inhibitors) OR (noradrenaline-dopamine reuptake inhibitor) OR (noradrenaline-dopamine reuptake inhibitors) OR (noradrenaline-dopamine re-uptake inhibitor) OR (noradrenaline-dopamine reuptake inhibitors) OR (norepinephrine and dopamine reuptake inhibitor) OR (norepinephrine and dopamine reuptake inhibitors) OR (norepinephrine and dopamine re-uptake inhibitor) OR (norepinephrine and dopamine re-uptake inhibitors) OR (bupropion) OR (dexmethylphenidate) OR (methylphenidate) OR (ndra) OR (nras) OR (norepinephrine dopamine releasing agent) OR (norepinephrine dopamine releasing agents) OR (norepinephrine-dopamine releasing agent) OR (norepinephrine-dopamine releasing agents) OR (norepinephrine and dopamine releasing agent) OR (norepinephrine and dopamine releasing agents) OR (norepinephrine dopamine releasing agent) OR (norepinephrine dopamine releasing agents) OR (noradrenaline-dopamine releasing agent) OR (noradrenaline-dopamine releasing agents) OR (norepinephrine and dopamine releasing agent) OR (norepinephrine and dopamine releasing agents) OR (amphetamine) OR (dextroamphetamine) OR (dextromethamphetamine) OR (lisdexamfetamine) OR (teca) OR (texas) OR (tetracyclic antidepressant) OR (tetracyclic antidepressants) OR (tetracyclic antidepressant) OR (tetracyclic anti-depressants) OR (amoxapine) OR (maprotiline) OR (mianserin) OR (mirtazapine) OR (maoi) OR (maois) OR (monoamine oxidase inhibitor) OR (monoamine oxidase inhibitors) OR (isocarboxazid) OR (moclobemide) OR (phenelzine) OR (pirlindole) OR (selegiline) OR (tranylcypromine) OR (antidepressant) OR (antidepressants) OR (antidepressant) OR (anti-depressants))))	#2
(forced swimming test OR forced swimming tests OR forced swimming test, fst OR fst OR forced swim OR porsolt test OR porsolt tests)	#3

**Table 2:** Keywords for search in the SCOPUS database

(rodentia OR mice) OR mus) OR mouse) OR murine) OR woodlouse) OR rats) OR rat) OR murinae) OR muridae) OR cottonian) OR cottonrat) OR rodentia) OR rodent) OR rodents)	#1
(ssri OR ‘selective serotonin-reuptake inhibitor’ OR fluoxetine OR citalopram OR escitalopram OR fluvoxamine OR paroxetine OR sertraline OR dapoxetine OR snri OR ‘serotonin and norepinephrine reuptake inhibitor’ OR duloxetine OR levomilnacipran OR sibutramine OR bicifadine OR venlafaxine OR desvenlafaxine OR milnacipran OR tramadol OR TCA OR ‘tricyclic antidepressant’ OR amitriptyline OR butriptyline OR clomipramine OR desipramine OR dosulepine OR doxepin OR imipramine OR iprindole OR lofepramine OR melitracen OR nortriptyline OR opipramol OR protriptyline OR trimipramine OR sari OR ‘serotonin antagonist and reuptake inhibitor’ OR etoperidone OR lorpiprazole OR mepiprazol OR lurasodone OR nefazodone OR trazodone OR nris OR ‘norepinephrine reuptake inhibitors’ OR atomoxetine OR reboxetine OR viloxazine OR ndri OR ‘norepinephrine dopamine reuptake inhibitor’ OR bupropion OR dexmethylphenidate OR methylphenidate OR ndma OR nras OR ‘norepinephrine dopamine releasing agent’ OR amphetamine OR dextroamphetamine OR dextromethylamphetamine OR lisdexamfetamine OR teca OR texas OR ‘tetracyclic antidepressant’ OR amoxapine OR maprotiline OR mianserin OR mirtazapine OR maoi OR ‘monoamine oxidase inhibitor’ OR isocarboxazid OR moclobemide OR phenelzine OR pirlindol OR selegiline OR tranylcypromine OR antidepressant)	#2
(‘forced swimming test’ OR ‘forced swimming tests’ OR ‘forced swimming test’ OR fst OR fst OR ‘forced swim’ OR ‘porsolt test’ OR ‘porsolt tests’)	#3

**Table 3:** Keywords for search in the Web of Science database

(rodentia OR mice) OR mus) OR mouse) OR murine) OR woodlouse) OR rats) OR rat) OR murinae) OR muridae) OR cottonian) OR cottonrat) OR rodentia) OR rodent) OR rodents)	#1
(ssri OR ‘selective serotonin-reuptake inhibitor’) OR fluoxetine) OR citalopram) OR escitalopram) OR fluvoxamine) OR paroxetine) OR sertraline) OR dapoxetine) OR snri) OR ‘serotonin and norepinephrine reuptake inhibitor’) OR duloxetine) OR levomilnacipran) OR sibutramine) OR bicifadine) OR venlafaxine) OR desvenlafaxine) OR milnacipran) OR tramadol) OR TCA) OR tricyclic antidepressant) OR amitriptyline) OR butriptyline) OR clomipramine) OR desipramine) OR dosulepine) OR doxepin) OR imipramine) OR iprindole) OR lofepramine) OR melitracen) OR nortriptyline) OR opipramol) OR protriptyline) OR trimipramine) OR sari) OR ‘serotonin antagonist and reuptake inhibitor’) OR etoperidone) OR lorpiprazole) OR mepiprazol) OR lurasodone) OR nefazodone) OR trazodone) OR nris) OR ‘norepinephrine reuptake inhibitors’) OR atomoxetine) OR reboxetine) OR viloxazine) OR ndri) OR ‘norepinephrine dopamine reuptake inhibitor’) OR bupropion) OR dexmethylphenidate) OR methylphenidate) OR ndma) OR nras) OR ‘norepinephrine dopamine releasing agent’) OR amphetamine) OR dextroamphetamine) OR dextromethylamphetamine) OR lisdexamfetamine) OR teca) OR texas) OR ‘tetracyclic antidepressant’) OR amoxapine) OR maprotiline) OR mianserin) OR mirtazapine) OR maoi) OR ‘monoamine oxidase inhibitor’) OR isocarboxazid) OR moclobemide) OR phenelzine) OR pirlindol) OR selegiline) OR tranylcypromine) OR antidepressant)	#2
(‘forced swimming test’ OR ‘forced swimming tests’ OR ‘forced swimming test’ OR fst OR fst OR ‘forced swim’ OR ‘porsolt test’ OR ‘porsolt tests’)	#3

### Inclusion criteria

The following inclusion criteria will be applied to the systematic review outcome:

- Publication date: since 1977, the year the first paper was published, to present (December 2017).
- Language: any language.
- Animal species: rats and mice, regardless of age and sex.
- Type of publication: all types of publications containing studies describing the effect of all classes of clinically tested antidepressant drugs in FST, compared with control animals treated or not with vehicle will be included, regardless of randomisation. The antidepressants included in this review will be dose listed in the protocol by McCann *et al*.^17^ In future studies, we intend to include publications containing information about candidate substances (plant-derived compounds such as polyphenols and terpenoids, and ketamine).
- Studies with any route, dose and treatment schedule for drug administration are eligible.

### Exclusion criteria

The following exclusion criteria will be applied to the inclusion criteria application outcome:

- Experiments using FST in rats or mice, only as a stressor, without showing the data of the behavioural measures.
- Experiments reporting data of co-treatments. The publications containing these experiments will be kept if they also report experiments with single treatments.

### Search strategy

Publications returned from the searches will be exported to a single reference manager file. Duplicate references will be deleted. Two investigators will independently evaluate the titles and abstracts obtained to assess if they meet the broad inclusion criteria and compare their results. If there is any discrepancy in included titles, consensus will be reached through discussion with a third investigator.

### Data extraction

Data on outcome measures (primary and secondary) and attributes of study quality (see items 3.6, 3.7 and 3.8) will be recorded. One investigator will carry out initial data extraction and a second investigator will then check all data entered. Primary outcome measures extracted will be the parameters (total or mean duration, percentage or punctuation) of immobility. Secondary outcome measures will be the parameters of active behaviours (swimming, climbing and headshakes) and index of locomotion (in the open field, or rotarod or another test).

### Design of study

Data on study design will be recorded, including: species, strain, age, weight and sex of animals used; number of experimental groups and number of animals per group; number of experiments and replications; housing conditions (food and water regimens, light cycle, temperature, size of the cage, length of housing the laboratory conditions); experimental conditions (time, illumination, dimensions of the tank, temperature and volume of water); FST protocol (single or repeated sessions, eg, only test, or pretest followed by one or more tests, length of swimming sessions); antidepressant subtype, dose (mg/kg) or regimen (single or multiple), mode of action of administered antidepressant; timing of drug administration related to the time point of the outcome measurement (test session of FST); methods of outcome measurement (manual or automatised); statistical methods for comparing groups and specific data from behavioural measures (mean, SD or SE of mean); reporting of data exclusion or inclusion for analysis.

### Quality of study

A checklist with 10 items adapted from CAMARADES^18 19^ will be used to assess the quality of experimental design. Additionally, a user-defined checklist will be used to assess the quality of the protocols of FST. The items in the user-defined checklist were chosen considering previous experience of the authors (eg, Lino-de-Oliveira *et al* and Mezadri *et al*,^6 7^ and other published literature, eg, Porsalt *et al*, Detke *et al* and Petit-Demouliere *et al* 4 5 21). Information in the user-defined checklist is relevant to reproducibility of the study. Publications will receive a point for compliance of each item in the checklist from which group median scores will be calculated.

Ten items in the checklist adapted from CAMARADES are: (1) Peer-reviewed publication. (2) Reporting species/strain of animals in the title or abstract and in the full text. (3) Statement of compliance with animal welfare regulations. (4) Use of animals with behavioural phenotype. (5) Reporting blinded assessment of outcome. (6) Statement of possible conflicts of interest. (7) Reporting randomisation of treatment allocation. (8) Reporting sample size calculation. (9) Reporting criteria for inclusion and/or exclusion of data. (9) Reporting concealment of treatment allocation.

Ten items in the user-defined checklist are: (1) Reporting sex of animals in the title or abstract or in the full text. (2) Reporting age or weight or phase of development of animals in the title or abstract or in the full-text reporting. (3) Reporting efforts for improving animal welfare (eg, environmental enrichment) and reducing the number of animals. (4) Reporting control of temperature and light phase in the animal house. (5) Reporting dimensions of the tank used in FST. (6) Reporting the volume or the height of the water in the tank. (7) Reporting the temperature of water used in FST. (8) Reporting the use of clean water in FST. (9) Control for impaired locomotion of animals. (10) Reporting method of behavioural measurements.

### Outcome extraction

The number of animals (N), the mean (M), SD or SE of the primary outcome (parameters of immobility) will be extracted for each treatment comparison. When available in the publications, N, M, SD or SE of secondary outcomes (ambulation in the open field test or active behaviours in FST) will be extracted. Ambulation in the open field test is used in many studies to discard influence of treatments on the motor function of animals (eg,^4 6^) while active behaviours in FST such as swimming and climbing may indicate mechanism of action of antidepressants in rats.^5^ In cases where the full data required for meta-analysis are not available in the text or tables in the publications, digital ruler software will be used to measure data from graphs. If information is completely unavailable in the publication, it will be requested directly from authors. When required data are not obtainable, such studies will be excluded from the analysis.

### Meta-analysis

Analytical choices were made from pilot studies published elsewhere.^13–15^ Normalised mean difference will be used to calculate ES. SE of ES will be calculated for each comparison. In addition, the 95% CI of the ES will be calculated. Data will be aggregated using a weighted average method in which greater weight is given to more precise studies. For anticipated heterogeneity between studies, the random-effects model of DerSimonian and Laird will be used, which is more conservative than fixed-effect models, given the weighting towards individual comparisons depends on the variance within those comparisons and on overall heterogeneity. The heterogeneity between studies will be assessed by using the χ^2^ statistic with n−1 degrees of freedom. To allow for multiple comparisons, a significance level will be set using Bonferroni correction taking into account the number of comparisons. Publication bias will be looked for using funnel plotting, Egger regression and ‘trim and fill’. Subgroup meta-analysis (meta-regression, and if necessary, stratified regression) will be performed when possible according to characteristics of study design and study quality to assess their impact on efficacy, as the following subgroups: species, strain, age, comorbidities (present/absent), FST protocol; antidepressant subtype, dose (mg/kg), regimen (single or multiple) and time of administration (relative to the test), time of outcome assessment (relative to the test), scores of quality checklist, especially the use of randomisation and blinded assessment of outcome.^18 19 22^

## Conclusion

Problems in reproducibility of preclinical studies are one of the major concerns in antidepressant research. Importantly, medical decisions are grounded on clinical trials and these are based on results reported in preclinical studies. Therefore, the existence of publication or confirmation risk of bias as well as low statistical power and method quality prevent from obtaining accurate evidence for developing therapeutic interventions. Hence, results obtained with the present protocol for conducting a systematic review with meta-analysis of preclinical studies using FST for testing antidepressant responses in rodents intends to improve the quality and statistical power of future studies and to contribute towards applying the 3R (**R**eplacement,  **R**eduction and  **R**efinement) principles in preclinical research.

## References

[1] Fried EI , Nesse RM . Depression sum-scores don’t add up: why analyzing specific depression symptoms is essential. BMC Med 2015;13:72. 10.1186/s12916-015-0325-4 25879936 PMC4386095

[2] Cryan JF , Valentino RJ , Lucki I . Assessing substrates underlying the behavioral effects of antidepressants using the modified rat forced swimming test. Neurosci Biobehav Rev 2005;29(4-5):547–69. 10.1016/j.neubiorev.2005.03.008 15893822

[3] Berton O , Durand M , Aguerre S , et al . Behavioral, neuroendocrine and serotonergic consequences of single social defeat and repeated fluoxetine pretreatment in the Lewis rat strain. Neuroscience 1999;92:327–41. 10.1016/S0306-4522(98)00742-8 10392854

[4] Porsolt RD , Bertin A , Jalfre M . Behavioral despair in mice: a primary screening test for antidepressants. Arch Int Pharmacodyn Ther 1977;229:327–36. 596982

[5] Detke MJ , Rickels M , Lucki I . Active behaviors in the rat forced swimming test differentially produced by serotonergic and noradrenergic antidepressants. Psychopharmacology 1995;121:66–72. 10.1007/BF02245592 8539342

[6] Lino-de-Oliveira C , De Lima TC , de Pádua Carobrez A . Structure of the rat behaviour in the forced swimming test. Behav Brain Res 2005;158:243–50. 10.1016/j.bbr.2004.09.004 15698890

[7] Mezadri TJ , Batista GM , Portes AC , et al . Repeated rat-forced swim test: reducing the number of animals to evaluate gradual effects of antidepressants. J Neurosci Methods 2011;195:200–5. 10.1016/j.jneumeth.2010.12.015 21167866

[8] Kara NZ , Stukalin Y , Einat H . Revisiting the validity of the mouse forced swim test: Systematic review and meta-analysis of the effects of prototypic antidepressants. Neurosci Biobehav Rev 2018;84:1–11. 10.1016/j.neubiorev.2017.11.003 29128579

[9] Carvalho AF , Cavalcante JL , Castelo MS , et al . Augmentation strategies for treatment-resistant depression: a literature review. J Clin Pharm Ther 2007;32:415–28. 10.1111/j.1365-2710.2007.00846.x 17875106

[10] Santaguida P , States U . Agency for Healthcare Research and Quality., McMaster University. Evidence-based Practice Center. Treatment for depression after unsatisfactory response to SSRIs. Comparative effectiveness review no 62. Rockville, MD: Agency for Healthcare Research and Quality, 2012:1.

[11] Belzung C . Innovative drugs to treat depression: did animal models fail to be predictive or did clinical trials fail to detect effects? Neuropsychopharmacology 2014;39:1041–51. 10.1038/npp.2013.342 24345817 PMC3957126

[12] Sena ES , van der Worp HB , Bath PM , et al . Publication bias in reports of animal stroke studies leads to major overstatement of efficacy. PLoS Biol 2010;8:e1000344. 10.1371/journal.pbio.1000344 20361022 PMC2846857

[13] Ramos-Hryb AB , Harris C , Aighewi O , et al . How would publication bias distort the estimated effect size of prototypic antidepressants in the forced swim test? Neurosci Biobehav Rev 2018;92:192–4. 10.1016/j.neubiorev.2018.05.025 29886179

[14] Lino de Oliveira C . A protocol for systematic review and meta-analysis of data from preclinical studies employing the forced swimming test: CAMARADES, 2016. 10.1136/bmjos-2017-000043 PMC8749270 35047683

[15] Lino de Oliveira C , Bahor Z , Currie G , et al . Search strategy for the protocol: A protocol for systematic review and meta-analysis of data from preclinical studies employing the forced swimming test. 26/02/2016 edn: CAMARADES, 2016. 10.1136/bmjos-2017-000043 PMC8749270 35047683

[16] Hooijmans CR , Tillema A , Leenaars M , et al . Enhancing search efficiency by means of a search filter for finding all studies on animal experimentation in PubMed. Lab Anim 2010;44:170–5. 10.1258/la.2010.009117 20551243 PMC3104815

[17] McCann SK , Irvine C , Mead GE , et al . Efficacy of antidepressants in animal models of ischemic stroke: a systematic review and meta-analysis. Stroke 2014;45:3055–63. 10.1161/STROKEAHA.114.006304 25184357

[18] Macleod MR , O’Collins T , Howells DW , et al . Pooling of animal experimental data reveals influence of study design and publication bias. Stroke 2004;35:1203–8. 10.1161/01.STR.0000125719.25853.20 15060322

[19] Jerndal M , Forsberg K , Sena ES , et al . A systematic review and meta-analysis of erythropoietin in experimental stroke. J Cereb Blood Flow Metab 2010;30:961–8. 10.1038/jcbfm.2009.267 20040929 PMC2949185

[20] de Vries RBM , Hooijmans CR , Langendam MW , et al . A protocol format for the preparation, registration and publication of systematic reviews of animal intervention studies. Evid Based Preclin Med 2015;2:e00007–9. 10.1002/ebm2.7

[21] Petit-Demouliere B , Chenu F , Bourin M . Forced swimming test in mice: a review of antidepressant activity. Psychopharmacology 2005;177:245–55. 10.1007/s00213-004-2048-7 15609067

[22] Sena ES , Currie GL , McCann SK , et al . Systematic reviews and meta-analysis of preclinical studies: why perform them and how to appraise them critically. J Cereb Blood Flow Metab 2014;34:737–42. 10.1038/jcbfm.2014.28 24549183 PMC4013765

